# The Effects of Temperature on the Development of the Moth *Athetis lepigone*, and a Prediction of Field Occurrence

**DOI:** 10.1673/031.013.10301

**Published:** 2013-10-06

**Authors:** Li-Tao Li, Yu-Qiang Wang, Ji-Fang Ma, Lei Liu, Yan-Tang Hao, Chao Dong, Yao-Jin Gan, Zhi-Ping Dong, Qin-Ying Wang

**Affiliations:** 1 Institute of Millet Crops, Hebei Academy of Agriculture and Forestry Sciences, Shijiazhuang, China; 2 Plant Protection Station of Xing Tai City, Xingtai, China; 3 Plant Protection Station of Guantao County, Handan, China; 4 College of plant protection, Agricultural University of Hebei, Baoding, China; # These authors contributed equally to this work

**Keywords:** developmental duration, developmental threshold temperature, effective accumulative temperature, generation, life table

## Abstract

*Athetis lepigone* (Möschler) (Lepidoptera: Noctuidae) is an important insect pest of corn crops in China. To determine the effect of temperature on *A. lepigone* growth, and to provide a forecasting model for this pest, the development and fecundity of *A. lepigone* under five different temperatures (18, 21, 24, 27, 30° C) was investigated, and an experimental population life table was constructed based on the obtained results. The results showed that the duration of development of *A. lepigone* decreased as the temperature increased from 18 to 30° C. Approximately 95% of mature larvae stopped pupating at 18° C, and about 70% of mature larvae stopped pupating at 21° C. When the growth chamber temperature was above 24° C, no growth arrest was observed. The results indicated that the optimum growth temperature of *A. lepigone* was about 26.47° C. In this study, the highest survival rate, fecundity per female, and population index trend were observed when the temperature was set at 27° C. The percentages of larvae that could spin cocoons after the 5th or 6th instar differed at the different temperatures. The developmental threshold temperatures for *A. lepigone* eggs, larvae, pre-pupae, pupae, preoviposition females, and the whole generation (i.e., egg to oviposition) were 11.03, 9.04, 15.08, 11.79, 11.63, and 10.84° C, respectively, and their effective accumulative temperatures were 63.51, 339.42, 30.04, 118.41, 35.06 and 574.08 degree-days, respectively. Based on the effective accumulative temperature law, this pest insect can have four generations in most of the Huang-Huai region of China, and two to three generations annually in some cold regions. *Athetis* *lepigone* may have four generations in the mid-southern part of Hebei Province. This prediction matches the field survey results.

## Introduction

*Athetis lepigone* (Lepidoptera, Noctuidae) (Möschler 1860) is known to occur in many European countries, east-central parts of Russia, Japan, North Korea, and Mongolia (Nowacki et al. 2001; Nikolaevitch et al. 2003; Lindeborg 2008; Poltavsky et al. 2009). It was first reported in Shenyang, China, in 1999 (Chen 1999; Zhang et al. 2009). Because *A*. *lepigone* was not known to cause severe damage to field crops, this insect was only briefly described ( Szőcs et al. 1981; Nieminen et al. 1998). However, *A. lepigone* has caused severe damage to maize crops in China in recent years, but its growth and life cycle characteristics have not been investigated.

In recent years, leaving plant straws in fields and no-tillage seeding have become common practices in China. This new cultivation system has created a suitable ecological environment and rich food source for *A*. *lepigone*. Consequently, since 2005 *A*. *lepigone* has become a major pest in summer corn fields in the Hebei Province. *Athetis* *lepigone* larvae drill and eat on the corn stems, resulting in the wilting and later death of the plant. It also chews on the aerial parts of corn roots, causing lodging and severe yield loss of corn crops (Jiang et al. 2008). A recent study by Zhu et al. (2012) indicated that, due to the changes in cultivation practices, the population of this pest insect in several provinces, including Shandong, Shanxi, Henan, Anhui, and Jiangsu, increased to a level of outbreak in 2011. In the fields with a serious occurrence of *A. lepigone*, the average number of larvae per corn plant ranged from 3 to 5, with maximum numbers of about 20 per plant (Jiang et al. 2008). Field surveys conducted in recent years indicated that *A*. *lepigone* had three significant emergence peaks (mid-June, mid to late July, and late August to early September) annually in the Hebei Province. The earliest emergence time for adult *A. lepigone* was in mid-April (Zhang et al. 2011).

Among the all known environmental factors, temperature is the main factor affecting insect growth and reproduction (Zhang 2002). Many studies were made to determine the relationship between temperature, development, and reproduction of agriculture pest insects. The results of these studies have provided useful information for predicting the occurrence of various pests in specific regions, predicting the number of generations each year, and designing control protocols ( Luo and Li 1993; Infante 2000; Fantinou et al. 2004; Feng et al. 2007; Kang et al. 2009; Ju et al. 2011).To elucidate the biological characteristics of *A. lepigone*, and to establish a forecasting model for the pest in China, the growth duration, the developmental threshold temperature, and the effective accumulative temperature for *A. lepigone* was investigated. An experimental population life table and a forecasting model are presented for this important pest.

## Materials and Methods

### Source of *Athetis epigone*

*Athetis lepigone* larvae were taken from corn fields in Shijiazhuang, Hebei Province, China, in July 2011, and kept indoors until emergence. Male and female moths were transferred together into insect cages and fed with 10% honey solution. The cages were covered with a layer of gauze during *A*. *lepigone* spawning. *A. lepigone* was grown inside a laboratory set at 26 ± 1°; C, 50%∼70% RH, and natural daylight. The progeny of the laboratory-reared *A. lepigone* were used in this experiment.

### Observation of developmental duration and data analysis

Over 150 newly-laid eggs were collected and treated with 5% formaldehyde for disinfection. After air-drying, the eggs were placed in plastic containers and incubated in growth chambers (Model E30B, Percival Scientific, www.percival-scientific.com) set at 18, 21, 24, 27 and 30 ± 0.5° C, respectively, and at 80 ± 5% RH and 14:10 L:D photoperiod. The total number of eggs hatched under each temperature was recorded, and the developmental duration of each egg (i.e., incubation period) was recorded.

After hatching, 90 larvae were placed individually in wells (3.5 × 1.5cm) of 6-well, flat-bottomed, tissue culture plates and were fed with fresh maize leaves until larvae matured. If the newly-emerged larvae had a high mortality rate at a temperature, the 1st instar larvae reared at 27° C were supplied to determine the development and survival of immature stages at this temperature. The headcapsule popping off was used to determine larval molting, and the complete formation of a cocoon was considered to be pre-pupae. In this study, the survival rate and the developmental duration of each growth stage were recorded. The survival rate was calculated as:

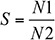
S is survival rate, N1 is the number of insects that progressed into the next developmental stage, and N2 is the number of insects in the previous developmental stage.

The eggs, larvae, pre-pupae, pupae, and adults were examined and recorded at three different times (8:30, 14:30, and 20:30) each day for their development progresses. The experiment was repeated three times.

In the adult stage, one newly emerged female and one newly emerged male (less than 24 hr old) were paired in an insect cage at each treatment temperature, fed with 10% honey solution, and this was replicated at least five times. Male and female adults were determined based on the appearance of the hair clusters at the ends of their bodies. The female hair cluster is open, and its gonopore is visible. The pleon end of an adult male is semitriangular, and its hair cluster is closed. The number of eggs laid under each temperature condition by each female was counted, and the pre-oviposition period, oviposition period, and longevity of adults were also recorded.

Temperature requirements and the rates of each developmental stage were calculated by using the results from the different treatments. The least squares method was used to calculate the developmental threshold temperature (C) and the effective accumulative temperature (K) for instars at various stages as described previously (Zhang, 2002). The following equations were used:

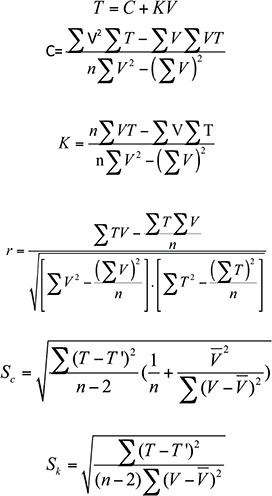
Where T represents the temperature, V is the developmental rate, r is the correlation coefficient, r is the sample number in the indicated temperature group, Sc is the standard error for the developmental threshold temperature, and Sk is the standard error for the effective accumulative temperature. Within the tested temperature range, the generation survival rate was parabolic. The relationship between the generation survival (Y) rate and temperature (T) was expressed as: Y= aT^3^ + bT^2^ + cT + d.

The experimental population life tables were formulated based on the effects of temperature on *A. 1epigone* survival, growth, development, and reproduction. The index of population trend was calculated by using the data shown in the life tables as described previously (Feng et al. 2007). Means and standard errors were determined by using the DPS version 7.05 software (Tang and Feng 2007), and the significant difference (*p* < 0.05) between different treatments was determined by using the single-factor analysis (one-way ANOVA), Duncan's multiple range test (*p* < 0.05), and correlation analysis (rvalue).

**Table 1.**
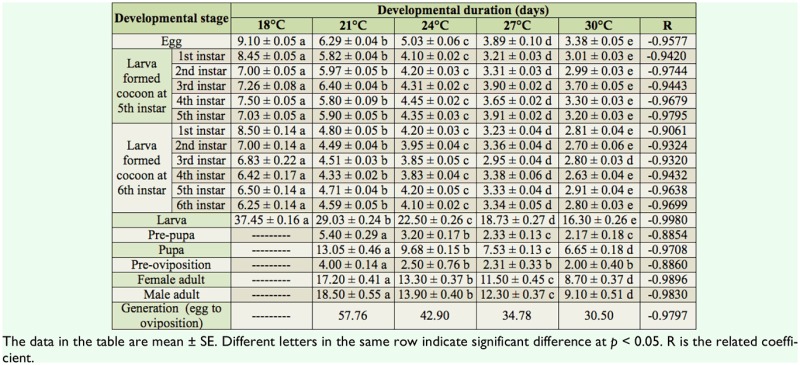
Developmental duration of *Athetis lepigone* at different temperatures.

**Table 2.**
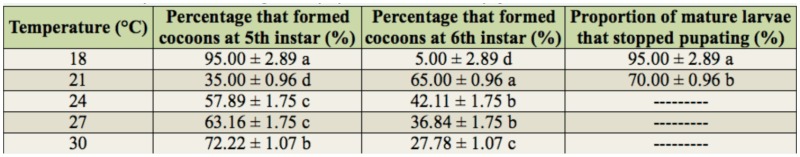
Influence of different temperatures on age and pupation of *Athetis lepigone*.

**Table 3.**
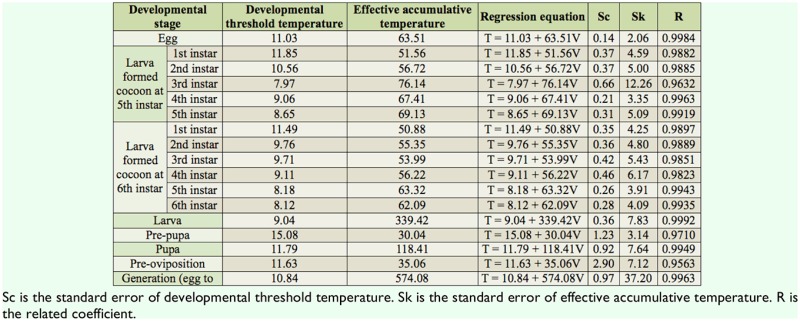
Developmental threshold temperature and effective accumulative temperature of *Athetis lepigone*.

### Observation of adult occurrence

*A. lepigone* adults were trapped by using the automatic pest forecasting lamps (', Hebi Jiaduo Science, www.jiaduo.net). The lamps were set up in fields in Guantao County. The observation period was from 1 April to 31 October 2011. The number of trapped adults was recorded daily, and the annual occurrence figure of *A. lepigone* adults was then prepared.

## Results

### Development of *Athetis lepigone* under different temperature conditions

The results of this study showed that temperature had significant effects on the developmental durations of *A. lepigone*. For the same developmental stages, the development duration significantly decreased as the temperature was increased from 18 to 30° C (Table 1) (eggs: *F*_4,595_ = 8145.2, *p* < 0.0001;for larvae spun cocoon after the 5th instar: 1st instar: *F*_4,176_ = 3223.9, *p* < 0.0001; 2nd in-star: *F*_4,176_ = 1771.3, *p* < 0.0001; 3rd instar: *F*_4, 176_ = 1327.8, *p* < 0.0001; 4th instar: *F*_4, 176_ = 1905.6, *p* < 0.0001; 5th instar: *F*_4. 176_ = 1712.8, *p* < 0.0001; for larvae spun cocoon after the 6th instar: 1st instar: *F*_4, 110_ = 654.0, *p* < 0.0001; 2nd instar: *F*_4, 110_ = 341.0, *p* < 0.0001; 3rd instar: *F*_4, 110_ = 418.1, *p* < 0.0001; 4th instar: *F*_4, 110_ = 237.7, *p* < 0.0001; 5th instar: *F*_4, 110_ = 302.1, *p* < 0.0001; 6th instar: *F*_4, 110_ = 316.3, *p* < 0.0001; larvae: *F*_4, 291_ = 4192.7, *p* < 0.0001; pre-pupae: *F*_3, 231_ = 160.2, *p* < 0.0001; pupae: *F*_3, 223_ = 782.5, *p* < 0.0001; pre-oviposition: *F*_3, 16_ = 5.3, *p* < 0.01; female adult: *F*_3, 16_ = 78.7, *p* < 0.0001; male adult: *F*_3, 16_ = 71.2, *p* <0.0001;).

The results shown in Table 2 indicate that the instar number of *A. lepigone* was influenced by different temperatures. It also indicates that the *A. lepigone* larvae could have 5 or 6 instars before pupation, and the percentages of 5 and 6 instar larvae varied under the different temperatures. For example, the highest percentage of larvae that formed cocoons after the 5th instar was observed at 18° C, and the highest percentage of larvae that formed cocoons after the 6th instar was observed at 21° C. The percentage of larvae that formed cocoons after the 5th instar increased when the temperature was increased from 21° C to 30° C. Approximately 95% of the population failed to complete their life cycle at 18° C and most of them stopped pupating in the cocoons. When the temperature was increased to 24° C or above, all larvae developed successfully into the adult stage.

### Developmental threshold temperature and effective accumulative temperature of different developmental stages

The developmental threshold temperatures of *A. lepigone* eggs, larvae, pre-pupae, pupae, and pre-oviposition ranged from 9.04 to 15.08° C, and the effective accumulative temperatures were from 30.04 to 339.42 degree days (Table 3). In the larval stage, the developmental threshold temperature was highest for 1st instar larvae (11.85 and 11.49° C). For all developmental stages, the developmental threshold temperature was highest for prepupae (15.08° C). The developmental threshold temperature for the entire generation was 10.84° C, and the effective accumulative temperature of the entire generation was 574.08 degree days (Table 3).

### The effect of temperature on *Athetis lepigone* survival

**Figure 1.**
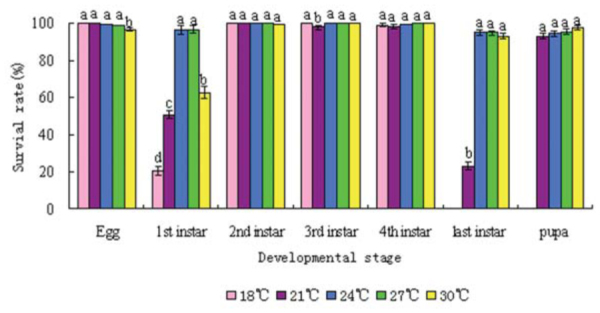
Survival rates of Athetis lepigone at different temperatures. High quality figures are available online.

The effects of temperature on the survival of *A. lepigone* varied at different developmental stages. Larvae at the 1st and last instar stages were more susceptible to the influence of temperature. The survival rate of 1st instar larvae was 20.56. 50.48. and 62.49% at 18,21, and 30° C, respectively (Figure 1). Survival rates increased to over 95% when the temperatures were 24 and 27° C. At 18° C, mature larvae stopped pupating. Within the temperature range of 21–30° C, the relationship between the generation survival (Y) and temperature (T) can be expressed as: Y =0.0914T^3^ +2.57 T^2^ +56.076T-1450.7. When the derivative was 0, 26.47° C was the optimum temperature.

### Experimental population life table of *Athetis lepigone*

The experimental population life tables were produced based on the survival rates of the different developmental stages and the adult fecundity data obtained from the different temperature treatments. The number of live eggs under different temperatures was assumed to be 100 (Table 4). The number of instars going into one stage was calculated according to the actual survival rates. The optimum temperatures for pupae and adult of *A*. *lepigone* were between 24 and 27° C. At 18° C, both pupae and adult *A. lepigone* stopped developing. No significant differences were observed between adult sex ratios under various temperature conditions. Fecundity was highest at 27° C (345.15 eggs per female) and showed approximately 17% reduction when the temperatures were reduced to 24 and 21° C, and 12.5% reduction when the temperature was increased to 30° C. The highest population trend index of *A. lepigone* was observed at 27° C, suggesting that under this condition *A. lepigone* can propagate rapidly and may result in an outbreak if food sources are available.

**Table 4.**
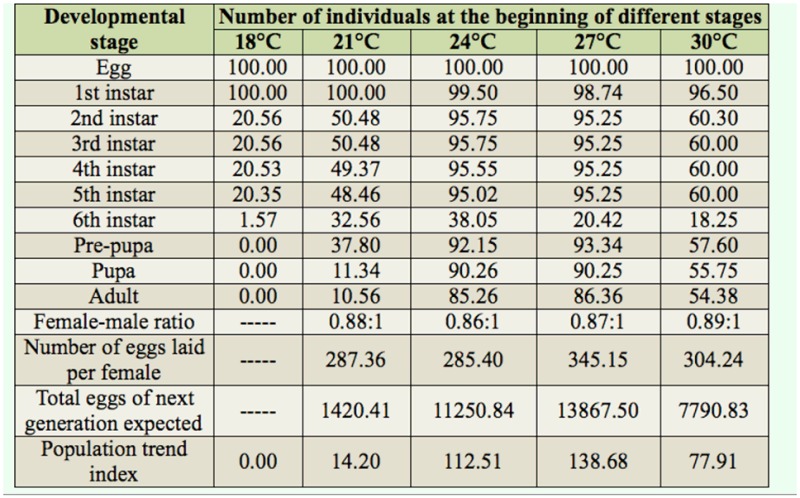
Experimental population life tables of *Athetis lepigone* at different temperatures.

**Figure 2.**
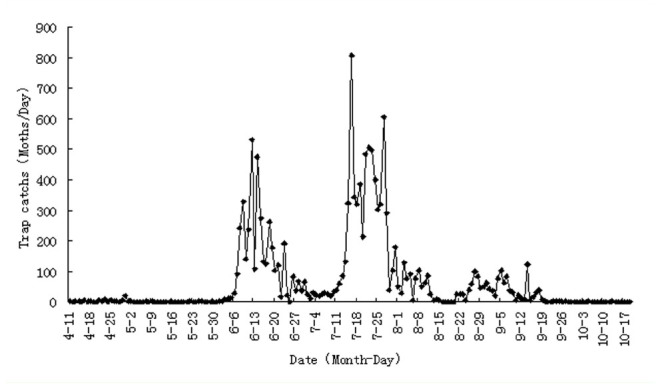
The daily amount of moths trapped by an insect killer lamp in Guantao County in 2011. High quality figures are available online.

### Estimated generation numbers of *Athetis* *lepigone* in different geographical regions each year

The data suggest that *A. lepigone* begins to develop when the temperature is above 10.84° C. Assuming the regional effective accumulative temperature is the sum of temperature above 10.84° C each month and throughout the year, the total effectiveaccumulative temperature in Guantao, Hebei Province, in 2011 was 2183.34 degree days. Because *A. lepigone* requires approximately 574.08 degree days to complete a whole generation cycle, it was proposed that *A*. *lepigone* could occur 3.80 generations in Guantao in 2011. Using the same method, it was propose that the estimated generations per year for Zhengding, Zhengzhou, Jinan, Bozhou, and Xuzhou would be 4.14, 4.01, 4.04, 4.20, and 4.16, respectively. For Shenyang, Chengde, Tangshan, and Jincheng, the estimated generation numbers are 2.67, 2.57, 3.51, and 3.23, respectively (Table 5).

As shown in Figure 2, adult *A. lepigone* (overwintered adults) appeared in early April 2011 in the fields of Guantao, and its population remained small until May. A small emergence peak was observed on 30 April. Three obvious emergence peaks were observed in 2011 in Guantao. During the first emergence peak period (mid-June), the number of moths captured by using the insect trapping lamps ranged from 100 to 523 per day. The second peak period was observed between mid and late July, and the largest number of moths captured was 806 per day.

**Table 5.**
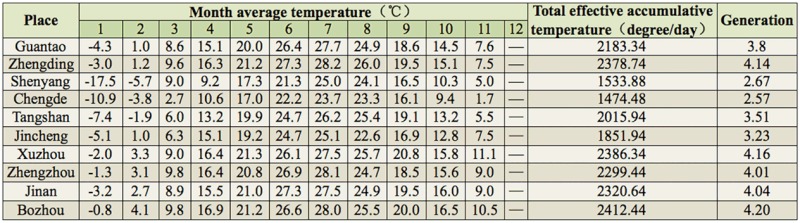
Monthly average temperatures and theoretical generations in different places in 2011. Data is from the weather bureau.

**Table 6.**
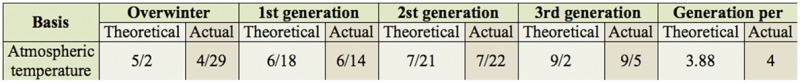
The theoretical date, the actual occurrence, and the annual generations of *Athetis lepigone* adults in Guantao.

The third peak period occurred in late August until early September, with less than 125 moths captured per day. This observation indicates that *A. lepigone* can have four generations per year in Guantao.

To predict the peak period of adult *A. lepigone* of each generation, the effective accumulative temperature was calculated using the local average temperature provided by the meteorological department. The laboratory observations indicated that, after overwintering, mature larvae could start their pupation just 1 day after incubation at room temperature (data not published). The meteorological data collected in 2011 showed that the daily mean temperatures of 6 days in March were above the developmental threshold temperature for pre-pupae, and 4 of the 6 days had temperatures above 20° C . If the emergence peak of the winter generation adults was predicted using the pupae developmental threshold temperature and the average daily temperature, the number of theoretical *A. lepigone* generations in Guantao per year would be 3.88 (Table 6). Using the average monthly temperature provided by the local meteorological department, the theoretical generation number inGuantao would be 3.80, similar to that predicted using the average daily temperature. The predicted adult emergence peaks, using the local temperature data, were on 2 May, 18 June, 21 July, and 2 September for the overwintering, first, second, and third generations, respectively. Thus, the prediction agrees with the field survey results.

## Discussion

The results of this study show that the developmental threshold temperature for different developmental stage *A. lepigone* was above 10° C, with the optimum temperatures ranging from 21 to 30° C. Within this temperature range, the developmental duration of each instar decreased as the temperature was increased, indicating a significant negative correlation between the temperature and the developmental duration. Both high and low temperatures had adverse effects on the survival of *A. lepigone* larvae and the fertility of adults. In addition to the tested temperatures in this experiment, the effect of high temperature on the mortality of larvae and adults was tested. The results showed that the newly hatched larva and adults had a higher mortality rate when the temperature reached above 33° C, indicating that *A*. *lepigone* was sensitive to high temperatures (Ma et al. 2012a). This observation is consistent with previously published results (Infante 2000; Ma et al. 2004; Ju et al. 2011; Li et al. 2011a). For example, *Corythucha ciliate* eggs did not hatch at 16° C, and the survival rate of eggs until the adult stage was lowest at 19° C. In addition, most nymphs did not develop further at 36° C (Ju et al. 2011).

It was reported that *A. lepigone* larvae had 5 (Li et al. 2011b) or 6 instars (Bai et al., 2012). However, we have shown here that the number of instars of *A. lepigone* was variable, and both 5 and 6 instars could be observed under the same temperature. The percentages of 5th or 6th instar larvae were influenced by temperature. Fan (2008) and others reported that male *Pristophora conjugata* larvae had 5 instars and female *P. conjugata* larvae had 6 instars. Under the same growth conditions, *Euproctis pseudoconspersa* larvae from different geographical populations had different numbers of larval instars (Yin et al. 2011). In our study, the sex and the source of population did not affect the number of larval instars. The significance of having different numbers of larval instars, other factors that determine *A*. *lepigone* growth (e.g., photoperiod and nutrition), and which larval instar is the most susceptible to the external influence require further investigation.

Many insects alter their developmental rate, or even suppress a particular developmental stage, to cope with environmental conditions differing from place to place and/or time to time (Danks 2002). Several studies have examined the developmental responses to environmental conditions, especially photoperiod and temperature (Li et al. 2008; Wang et al. 2009). Our study showed that temperature had a significant influence on thedevelopment of *A. lepigone*, causing some larvae to stop pupating in their cocoons during maturation at 18° C and 21° C. Low temperature was the inducing factor for *A. lepigone* dormancy (or diapause). In addition to the detailed developmental responses to temperature, the influence of photoperiod on *A. lepigone* development is currently under the investigation.

Each year, during the overlapping growing season between the wheat and corn crops in the Huang-Huaihai area of China, large amounts of wheat straw and stubble are left in the fields, and these leftover materials create a favorable environment for *A. lepigone*. In addition, newly-planted summer corn seedlings provide *A. lepigone* with sufficient food sources. Under suitable temperature conditions, *A. lepigone* larvae grow well and mature quickly, and the adults are often more fertile, leading to rapid reproduction and then an outbreak of the pest within a short period of time in June, the month with an average temperature of 26° C, in the south-central part of the Hebei Province (Jiang et al. 2008).

Due to irregular developmental patterns, some mature *A. lepigone* larvae do not produce cocoons, and some pupae survive during the warm winters in some regions. These surviving mature larvae and pupae often represent part of the first generation for the following year (Ma et al. 2012b). The developmental threshold temperature of prepupae is estimated to be 15.08° C. Based on the daily average temperatures in Guantao, it was estimated that the emergence peak of the overwintered *A. lepigone* would occur in late May. The actual field survey showed, however, that the overwintered *A. lepigone* adults first appeared in early April and reached the emergence peak in late April in 2011. The possible explanation for this earlier field emergence peak is that the accumulative temperature of the overwintered generation met the requirement sooner than had been predicted. The weather information obtained indicated that the daily highest temperatures in late May often exceeded 15.08° C. Consequently, a short period of warmer weather had led *A. lepigone* larvae to pupate sooner than had been predicted. Because the developmental threshold temperature for pupae is lower than that for the other developmental stages, the adult *A. lepigone* captured in April might have emerged from the overwintered pupae. Therefore, the prediction of the emergence peak of the overwintered adults, based on the developmental threshold temperature for pupae, agreed with the actual field survey results.

The predicted number of generations per year using the developmental threshold temperature and the effective accumulative temperature was similar to that observed in the field using insect trapping lamps. Although the developmental threshold temperature and the effective accumulative temperature presented in this paper were obtained under constant temperature conditions, and the variable temperatures in the field were reported to be more favorable for insect development and reproduction (Cai et al. 2001; Mironidis and SavopoulouSoultani 2008), the prediction of *A. lepigone* emergence peaks are still useful. It is noteworthy *A. lepigone* often survives inside the leftover plant materials in the field, which provide environmental conditions that are quite different from the conditions reported by meteorological data. In addition, the law of effective temperature emphasizes only the effect of temperature on development. Other factors, including food sources, can also have a significant impact on insect development duration, longevity, and fecundity (Sison and Shanower 1994; Shanower et al. 1997; Jalali et al. 2010; Saeed et al. 2010). Thus, feeding characteristics should also be considered when predicting the emergence peaks of *A. lepigone* using this method (Zhang 2002). Different generations of *A. lepigone* have different food sources. The impacts of different food sources on *A. lepigone* growth remain unknown and require further investigation.

The results showed that the *A. lepigone* generation number, the emergence peaks in a given year, and the duration of individual developmental stages could be predicted using the developmental threshold temperature, effective accumulative temperature, and temperature data provided by the local meteorological departments. This prediction provides useful information on *A. lepigone* occurrence dynamics and control strategies. According to the field surveys made in recent years, *A. lepigone* occurred in four generations annually in the south-central part of Hebei Province and often caused severe damage to the summer maize crops in late June until July. Overlapping between *A*. *lepigone* generations was seen frequently in field. The overwintered *A. lepigone* larvae could develop irregularly, resulting in small numbers of adult *A. lepigone* observed in the field in April of the following year (Ma et al. 2012c). The first, second, and third generation adults produced three emergence peaks each year (mid-June, mid to late July, and late August until early September). The number of adults captured during the first and second emergence peaks was significantly higher than that captured during the third peak period, with a daily maximum number of more than 1,000. Our investigation showed that *A*. *lepigone* was distributed unevenly in the field. For example, adults and larvae often stayed in shade to avoid significant temperature changes caused by direct sun light (Ma et al. 2012c). Although no report is available on *A*. *lepigone* damage to the maize crops in the northern part of Hebei Province, this insect was captured previously in Shenyang area using insect killer traps (Zhang 2009). *A*. *lepigone* larvae are known to be omnivorous. Indoor feeding surveys showed that this insect could eat leaves, stems, fruits, and even dry plant materials of more than 30 plant species belonging to 13 different families. These plant species include maize, wheat, soybean, peanut, sweet potato, cabbage, tomato, spinach, amaranth, goosefoot, purslane, and more (Ma et al. 2012a). Field investigations also indicated that *A. lepigone* larvae were able to feed on maize, wheat, sweet potato, soybean, peanut, and several vegetable species (Ma et al. 2012c). Although no report was available on *A. lepigone* damage to these field crops, it is obvious that favorable environments for this pest are widely present. Close attention should be paid to the population dynamics of this pest in the field.
